# Effects of Unfermented and Fermented Whole Grain Rye Crisp Breads Served as Part of a Standardized Breakfast, on Appetite and Postprandial Glucose and Insulin Responses: A Randomized Cross-over Trial

**DOI:** 10.1371/journal.pone.0122241

**Published:** 2015-03-31

**Authors:** Daniel P Johansson, Isabella Lee, Ulf Risérus, Maud Langton, Rikard Landberg

**Affiliations:** 1 Department of Food Science, BioCenter, Swedish University of Agricultural Sciences (SLU), Uppsala, Sweden; 2 Department of Public Health and Caring Sciences, Clinical Nutrition and Metabolism, Uppsala University, Uppsala, Sweden; 3 Nutritional Epidemiology Unit, Institute of Environmental Medicine, Karolinska Insitutet, Stockholm, Sweden; Louisiana State University, UNITED STATES

## Abstract

**Background:**

Whole grain rye products have been shown to increase satiety and elicit lower postprandial insulin response without a corresponding change in glucose response compared with soft refined wheat bread. The underlying mechanisms for these effects have not been fully determined The primary aim of the study was to investigate if whole grain rye crisp bread compared to refined wheat crisp bread, elected beneficial effects on appetite and postprandial insulin response, similarly as for other rye products.

**Methods:**

In a randomized cross-over trial, 23 healthy volunteers, aged 27-70 years, BMI 18-31.4 kg/m^2^, were served a standardized breakfast with unfermented whole grain rye crisp bread (uRCB), fermented whole grain rye crisp bread (RCB) or refined wheat crisp bread (WCB), Appetite was measured using a visual analogue scale (VAS) until 4 h after breakfast. Postprandial glucose and insulin were measured at 0-230 min. Breads were chemically characterized including macronutrients, energy, dietary fiber components, and amino acid composition, and microstructure was characterized with light microscopy.

**Results:**

Reported fullness was 16% higher (P<0.001), and hunger 11% and 12% lower (P<0.05) after ingestion of uRCB and RCB, respectively, compared with WCB. Postprandial glucose response did not differ significantly between treatments. Postprandial insulin was 10% lower (P<0.007) between 0-120 min but not significantly lower between 0-230 min for RCB compared with WCB. uRCB induced 13% (P<0.002) and 17% (P<0.001) lower postprandial insulin response between 0-230 min compared with RCB and WCB respectively.

**Conclusion:**

Whole grain rye crisp bread induces higher satiety and lower insulin response compared with refined wheat crisp bread. Microstructural characteristics, dietary fiber content and composition are probable contributors to the increased satiety after ingestion of rye crisp breads. Higher insulin secretion after ingestion of RCB and WCB compared with uRCB may be due to differences in fiber content and composition, and higher availability of insulinogenic branched chain amino acids.

**Trial Registration:**

ClinicalTrials.gov NCT02011217

## Introduction

Obesity and overweight are becoming more prevalent and causing increasing concern in large parts of the world [1]. Lifestyle factors such as low physical activity and poor diet are strong contributors to the development of obesity and its related diseases and are therefore important targets in the prevention and treatment of these conditions [2,3]. One approach is to reduce the overall energy intake by increasing the feeling of fullness and reducing hunger through tailored foods [4]. This is however complex, since appetite is controlled by both psychological factors and physiological responses related to food properties such as composition, energy density, and microstructure [5].

Products based on whole grain rye, mainly porridge and soft bread, have repeatedly been shown to induce higher subjective satiety [6–13] and lower postprandial insulin secretion with- or without corresponding difference in postprandial glucose profile [10,12–16] compared with refined wheat bread. Lower postprandial insulin response may increase insulin sensitivity and decrease low-grade inflammation and is therefore most likely beneficial for the prevention of Type 2 diabetes [17,18]. Several studies have attempted to explain the phenomenon of lower postprandial insulin responses without lower glucose responses, but it has not yet been fully explained. Specific features of rye products, such as dense structure and the formation of an amylose layer surrounding the starch granules [15], as well as certain phenolic acids found in some rye varieties [10], have been suggested as important for the beneficial postprandial insulin response. Furthermore, some amino acids, such as branched-chain amino acids (BCAA), lead to higher glucose-independent insulin secretion after a meal [19]. Lower concentrations of certain BCAA in blood plasma have also been demonstrated in trials, both after an eight week intervention and after a single meal with whole grain rye compared with refined wheat [20,21]. Processing can alter the microstructure, composition, and availability of different compounds and could be used to modulate the postprandial responses to a food. For example, fermentation has been shown to influence both the content and composition of dietary fiber (DF) [22] and the levels of some bioactive compounds [23] in rye products and flour.

In most studies to date on whole grain rye products such as porridge and soft bread, refined wheat bread has been used as the control product. Whole grain rye porridge and soft bread typically contain more water than soft, refined wheat bread, which gives lower energy density and may influence postprandial responses [24,25]. However, in a recent study we found that whole grain rye crisp bread, a product with low water content, promoted higher satiety, lower desire to eat, and lower hunger as well as lower energy intake in a subsequent meal compared with a soft, refined wheat bread [26]. A control product that is more similar in macrostructure and energy density may further reduce the influence of confounding factors.

The aim of the present study was to evaluate whether whole grain rye crisp bread, compared with refined wheat crisp bread, exerted beneficial effects on appetite and postprandial insulin response, as reported for other rye products. A further aim was to evaluate the effect of yeast fermentation by including one yeast fermented and one unfermented rye crisp bread as part of a breakfast.

## Materials and Methods

### Ethical statement

The study was carried out in agreement with the Helsinki Declaration and was approved by the regional ethics board in Uppsala on August 14, 2013 (dnr 2013/201). Recruitment started immediately after approval by the ethics board and the last participant was enrolled on August 27. The experimental work was completed by September 20, 2013. Individuals interested in participating were given written and oral information about the study and made aware of their option to withdraw from the study at any time they desired. Prior to inclusion in the study, written consent was obtained from all participants.

The study was registered at ClinicalTrials.gov (NCT02011217) after completion. The delay was due to unawareness by investigators regarding time frame for trial registration. The authors confirm that all ongoing and related trials for this drug/intervention are registered. The protocol for this trial and completed CONSORT checklist are available as supporting information, see S1 Protocol and S1 CONSORT Checklist.

### Participants

Participants for the study were recruited through an advertisement in a local newspaper, through a website for clinical trials advertisements, on the Ultuna campus of the Swedish University of Agricultural Sciences (SLU), and through an internal e-mail to employees at SLU. After initial screening, men and women who met the eligibility criteria were invited to take part in the study. These criteria to were: age 18–70 years, BMI 18.5–30 kg/m^2^, physical activity level <2, fasting plasma glucose <6.0 mmol/L, fasting serum insulin <11 mE/L, serum TSH <2.5 mIE/L, plasma LDL <5.3 mmol/L, fasting plasma triglycerides (p-TG) <1.8 mmol/L, non-smokers, no medications likely to affect appetite, no medical conditions involving the gastrointestinal tract, no gluten intolerance or food allergies, no physical problems with eating, habitual consumption of breakfast, lunch and dinner, no dieting or self-reported fluctuations in body weight of more than 10% three months prior to screening, no recent participation in a dietary study, and no pregnancy, lactation or intention to become pregnant during the study period.

### Study design

The study was conducted as a randomized, crossover trial with three different isocaloric breakfast treatments consisting of crisp breads served as part of a complete breakfast (Table 1). Test meal sequences followed a Latin square design and participants were randomly assigned, using the Excel function “Random”, to start one of the three treatments. Three commercially available crisp breads were used in the study: unfermented whole grain rye crisp bread (uRCB), yeast-fermented whole grain rye crisp bread (RCB) and yeast-fermented refined wheat crisp bread (WCB), (Barilla Sweden AB, Sweden). Flour of the same origin and composition but milled to different particle sizes was used for the production of uRCB and RCB. According to data provided by the manufacturer 30–42% of particles in the flour used for uRCB were below 125 μm and 20–28% above 1040 μm, while the same fractions in the flour used for RCB comprised 51–57% and 3–6% respectively. With the exception of particle size and the yeast added to RCB, the composition of uRCB and RCB was the same. The crisp breads were served with margarine and cheese, a glass of orange juice and a cup of coffee or tea (Table 1). Participants (Table 2) could choose between coffee and tea but had to adhere to their initial choice on all occasions. They were asked to refrain from eating, drinking and strenuous physical activity 12 hours prior to the test occasion, and to exclude alcohol and foods rich in dietary fiber from their diet per dietary guidance 24 hours before each test occasion. On the test day, participants arrived at the clinic at Uppsala University Hospital at least 10 minutes before the first appetite registration, taken at 9 am, which was 30 minutes before breakfast. Participants were instructed to finish the breakfast within 15 minutes. The test ended four hours after breakfast and participants stayed at the clinic the whole time. During this time the participants were not allowed to eat or drink anything not included in the study diet and were instructed to maintain a low physical activity. Conversation was permitted, except to discuss food or the study or to compare satiety ratings. Between each test occasion, there was a wash-out period of at least six days.

**Table 1:** Food and nutrient composition of the breakfasts used in the study^1^.

	Amount	Nutrient (g)/energy (kJ) per portion				
	(g)	Fat	Protein	CHO	Fiber^2^	Energy^3^
			uRCB			
Crisp bread	58.5	1.2	5.3	36.4	11.3	844
Margarine	15	5.9	0.1	0.2	0	223
Cheese	20	5.8	4.8	0	0	296
Orange juice	100	0.1	0.7	9.0	0.7	174
***Total***	193.5	13	10.9	45.6	12.0	1538
***E (%)***		***31***	***12***	***50***	***6***	***100***
			RCB			
Crisp bread	60	1.3	5.5	38	10.2	867
Margarine	15	5.9	0.1	0.2	0	223
Cheese	20	5.8	4.8	0	0	296
Orange juice	100	0.1	0.7	9.0	0.7	174
***Total***	195	13.1	11.1	47.2	10.9	1561
***E (%)***		***31***	***12***	***51***	***6***	***100***
			WCB			
Crisp bread	52	4	6.3	34.7	2.9	867
Margarine	15	5.9	0.1	0.2	0	223
Cheese	20	5.8	4.8	0	0	296
Orange juice	100	0.1	0.7	9.0	0.7	174
***Total***	187	15.8	11.9	43.9	3.6	1561
***E (%)***		***37***	***13***	***48***	***2***	***100***

^1^150 mL of coffee or tea was included in all meals;

^2^Fiber content as analyzed by the Uppsala method with inclusion of fructans.

^3^Energy content was calculated using a conversion factor of 37 kJ/g for fat, 17 kJ/g for proteins and CHO and 8 kJ/g for fiber.

**Table 2:** Age, weight and BMI of participants who completed the study. Given as mean and standard deviation.

	Men (n = 7)	Women (n = 16)	All
Age (y)	59.1±14.7	60.6±11.0	60.1±12.1
Weight (kg)	70.8±7.5	67.0±11.5	68.3±10.3
BMI (kg/m2)	22.8±1.1	24.3±4.1	23.8±3.4

### Chemical analysis of foods

Crisp breads were milled with a cyclone sample mill (Retsch, Haan, Germany). Extractable and unextractable DF content and composition were analyzed according to the Uppsala method [27]. β-glucan was analyzed using a K-BGLU kit (Megazyme, Bray, Ireland) [28] and fructan content using a K-FRUC kit (Megazyme, Bray, Ireland) [29] (Table 3). Content of arabinoxylan and arabino-galactan was calculated assuming an arabinose/galactose ratio of 0.69 in extractable arabinogalactan [30]. Total fat, protein (Table 1) and amino acid composition (Table 4) were analyzed by a certified commercial testing laboratory (Eurofins, Lidköping, Sweden) using the Schmid-Bondzynski-Ratzlaff method [31], the Kjeldahl method with conversion factor 6.25 and ISO 13903:2005/13904:2005 respectively.

**Table 3:** Dietary fiber content and composition of unfermented rye crisp bread (uRCB), yeast fermented rye crisp bread (RCB) and yeast fermented refined wheat crisp bread (WCB) (% of dry matter).

Dietary fiber component		uRCB	RCB	WCB
Dietary fiber				
	total^a^	20.5	18.3	6.0
	extractable^b^	7.9	7.2	1.6
	unextractable	12.6	11.1	4.4
Arabinoxylan^c^				
	Total	8.8	8.6	2.5
	extractable	2.6	3.0	0.7
	unextractable	6.3	5.6	1.8
Arabino-galactan^c^		0.1	0.2	0.2
β-glucan		2.5	2.1	0.3
Cellulose and resistant starch^d^		2.7	2.5	1.4
Fructan		4.0	2.6	0.4
Klason lignin		1.3	1.3	0.5

^a^Calculated as the sum of fructan and total dietary fiber as analyzed by the Uppsala method [27].

^b^Calculated as the sum of fructan and total extractable dietary fiber as analyzed by the Uppsala method [27].

^c^Calculated from the sum of arabinose, xylose and galactose assuming an arabinose to extractable galactose ratio of 0.69 in arabino-galactan [30].

^d^Calculated as the difference between total β-glucan and glucose residues as analyzed by the Uppsala method [27].

**Table 4:** Amino acid composition of the three crisp breads.

Amino acid	uRCB	RCB	WCB
mg/serving crisp bread*			
Alanine	228	234	198
Arginine	287	270	255
Aspartic acid	369	342	270
Cysteine	129	120	140
Glutamine	1223	1200	2059
Glycine	246	258	239
Histidine	123	120	135
Isoleucine	193	210	250
Leucine	339	354	432
Lysine	187	162	140
Methionine	82	72	83
Phenylalanine	240	246	307
Proline	474	456	686
Serine	246	252	312
Threonine	176	186	172
Tryptophan	59	59	63
Tyrosine	146	156	203
Valine	275	288	286

*A serving of crisp bread was 58.5 g, 60 g and 52 g for uRCB, RCB, and WCB, respectively.

### Subjective appetite rating

Subjective appetite ratings were recorded 30 min before breakfast, at breakfast and at 30, 60, 90, 120, 150, 180, 210, and 240 min after breakfast, using an electronic visual analogue scale (VAS). On arriving at the study center, each participant was provided with a Palm computer model z22 (Palm Inc., Sunnyvale, USA) which prompted the participants to answer three questions: “How hungry do you feel right now?”, “How full do you feel right now?” and “How strong is your desire to eat right now?”. The participants marked their answer on a line anchored at each end with an extreme answer to each question; “Not at all hungry”/“Extremely hungry”, “Not at all full”/“Extremely full” and “Extremely strong”/“Not strong at all”. The computer then translated the mark to a number between 0–100. This method has been shown to give results which are in agreement with the conventional 100 mm VAS [32–34].

### Physiological parameters

Venous blood was collected by trained nurses 15 minutes before breakfast and at 15, 35, 65, 95, 125, 185 and 230 minutes after breakfast. Blood samples were collected into ice cold tubes prepared with either potassium EDTA or lithium heparin (Sarstedt, Nümbrecht, Germany). Immediately after blood sampling 160 μl protease inhibitor cocktail (1 SigmaFast tablet (Sigma S8820) in 2.2 ml H2O + 5.5 μl 10 mM KR-62436(DMSO)) were added to the test tube to prevent breakdown of appetite related gut peptides and incretines, which may be analyzed later for other purposes. The samples were kept on ice and processed immediately. Blood plasma was separated from buffy coat and erythrocytes by centrifugation at 2500 g for 10 min (4°C), aliquoted into 2.0 mL screw cap micro tubes (Sarstedt, Nümbrecht, Germany) and immediately placed in a freezer at -20°C. Plasma samples were transferred and stored in a freezer maintaining a temperature of -80°C. Analyses of serum insulin and plasma glucose concentrations were performed by established routine methods at the certified laboratory of the Department of Clinical Chemistry at Uppsala University Hospital. Serum insulin was analyzed on a Roche cobas 8000 e602 module (Roche Diagnostics GmbH, Mannheim, Germany) and plasma glucose on an Architect c8000 and c16000 (Abbott, Abbott Park, Illinois, USA).

### Bright field microscopy

Samples were fixed with glutaraldehyde vapor for 12 h followed by 2 h fixation with OsO4 vapor. Samples were then washed twice in distilled water, dehydrated in a series of aqueous ethanol of increasing concentration and finally infiltrated and polymerized with Technovit 7100.

The embedded samples were cut into 1.5 μm thick sections with an ultra-microtome (Leica EM UC6, Leica, Austria). The sections were stained with Lugol’s solution which stains proteins yellow, and starch dark blue/violet. It also distinguishes between amylopectin rich areas (beige/brown), and amylose (blue). The stained sections were examined using a Nikon Eclipse Ni-U microscope and the images were captured with a Nikon Digital Sight DS-Fi2 camera and processed with the software NIS-Elements BR (Nikon Instruments Inc., New York, USA).

### Statistical analysis

Differences between treatments were evaluated with models appropriate for cross- over designs using PROC mixed in SAS version 9.3 (SAS Institute Inc, Cary, NC, USA). Effects of treatments were evaluated by two separate models [5]. In model 1, occasion, breakfast treatment, time (all measurement points after breakfast meal were included), time x treatment interaction and treatment x occasion interaction terms were included as fixed effects. Participant was entered as a random effect variable. In model 2, area under the curve (AUC) was used as the response variable and treatment, occasion, and the treatment x occasion interaction term were included as fixed dependent variable in the model. Participant was entered as a random factor. Total AUC was calculated for each participant and breakfast treatment, using the trapezoid rule. Differences in postprandial glucose and insulin responses were evaluated using the total area under the curve (AUC_t = 0–230min_) as well as for the first 120 min (AUC_t = 0-120min_). Response variables were log-transformed in order to approximate a normal distribution in cases where data were skewed as judged by a Shapiro-Wilks test with P<0.05. Reported values in curves are unadjusted mean unless otherwise stated. Treatment effects are reported as model-adjusted least square mean (LSM) ± standard error (SE). P-values for all main effects reported are based on models in which non-significant (P>0.10) interaction terms were removed. When a significant interaction term between treatment and time was found, t-tests were performed at each time point. P<0.05 was considered statistically significant for all tests, except for removal of interaction terms (P>0.10).

The primary outcome metric of the study was AUC for appetite ratings. With a power of 0.9 and a level of α 0.05 for a two sided test, 12 participants are sufficient to detect a 10% difference in mean appetite ratings over 4.5 hours in a comparison between two foods [35]. Secondary outcome metrics were AUC for postprandial plasma glucose and serum insulin concentration. For these, at least ten participants is needed to obtain sufficient statistical power [36].

## Results

### Participants

**Fig 1:**
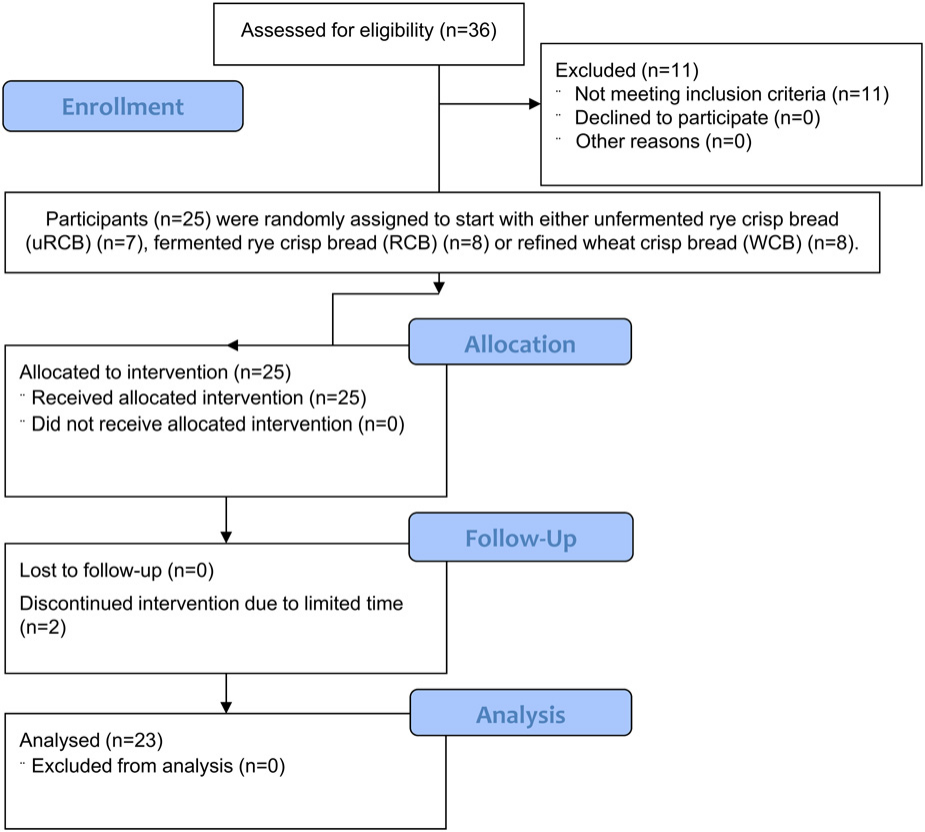
Flowchart of the study progress.

In total, 25 of the 36 participants screened were enrolled to participate in the study. Of these, three had BMI outside the range specified in the eligibility criteria but otherwise fulfilled the requirements and were deemed suitable for participation in the study. Two participants did not complete the study due to limited time. Thus a total of 23 participants completed the study and were included in the statistical evaluation. Participant’ age was 27–70 years and BMI 18–31.4 kg/m^2^ (Table 2). A flowchart of the study process is presented in Fig 1.

### Appetite ratings

**Fig 2:**
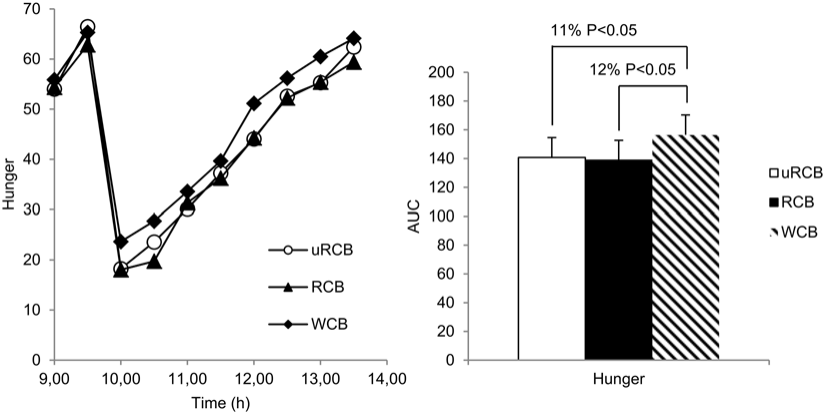
Subjective hunger. Left: Subjective hunger reported by n = 23 participants. Values are means. A statistically significant difference between treatments was found (P = 0.0043). Right: AUC for subjective hunger reported by n = 23 participants. A statistically significant difference between treatments was found (P = 0.0419). Values are adjusted least square means (LSM) ± standard errors (SE). Differences between treatments are given as percentage difference between the LSM values for AUC of the treatments. P<0.05 was considered significant.

**Fig 3:**
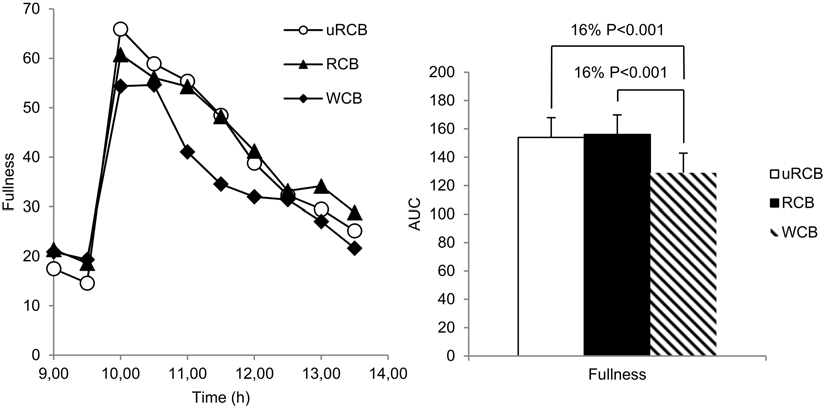
Subjective fullness. Left: Subjective fullness reported by n = 23 participants. Values are means. A statistically significant difference between treatments was found (P<0.0001). Right: AUC for subjective fullness reported by n = 23 participants. A statistically significant difference between treatments was found (P<0.0001). Values are adjusted least square means (LSM) ± standard errors (SE). Differences between treatments are given as percentage difference between the LSM values for AUC of the treatments. P<0.05 was considered significant.

**Fig 4:**
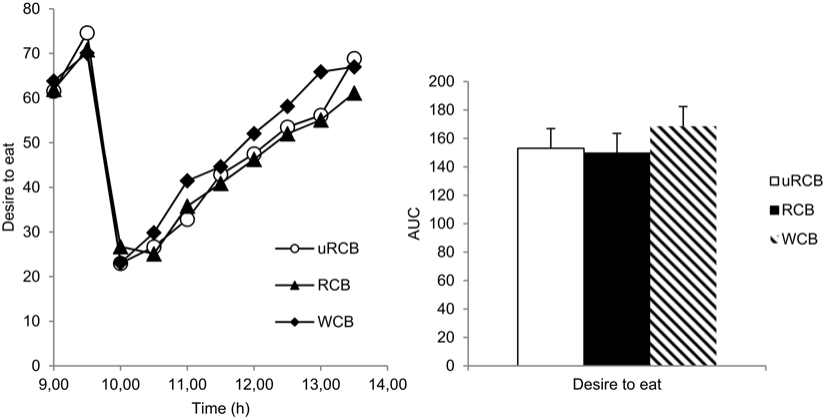
Subjective desire to eat. Left: Subjective desire to eat reported by n = 23 participants. Values are means. A statistically significant difference between treatments was found (P<0.0243). Right: AUC for subjective desire to eat reported by n = 23 participants. No statistically significant difference between treatments was found (P = 0.0832). Values are adjusted least square means (LSM) ± standard errors (SE).

For hunger, a significant difference between treatments was found (model 1: P = 0.004; model 2: P =. 0419). Hunger was 11% (P<0.05) lower for uRCB and 12% (P<0.05) lower for RCB compared with WCB (Fig 2). For fullness, there was a significant difference between treatments (model 1: P<0.0001; model 2: P<0.0001) (Fig 3). Fullness was 16% (P<0.001) higher for both rye crisp breads compared with WCB. For desire to eat, there was a significant difference between treatments when evaluating using model 1 (P<0.024) but not when using model 2 (AUC, P = 0.083) (Fig 4). There was no significant difference in any of the appetite ratings between uRCB and RCB.

### Postprandial glucose and insulin responses

**Fig 5:**
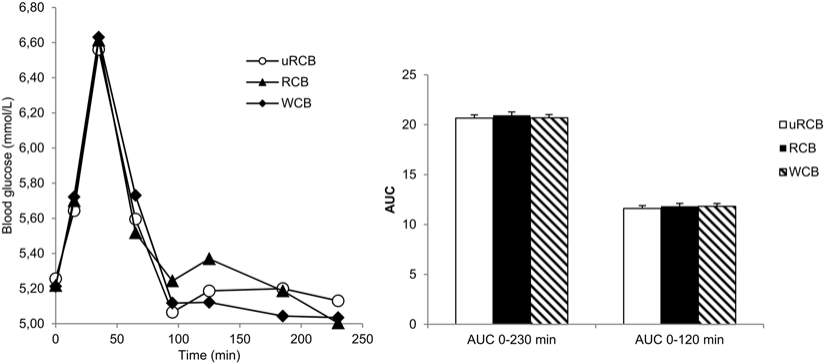
Plasma glucose profile and AUC. Left: Plasma glucose concentration- time profile in n = 23 participants. Values are means. No statistically significant effect attributed to breakfast treatment (P = 0.60) was obtained. Right: Differences in total AUC_0–230 min_ and AUC_0-120_ between breakfast treatments. Values are adjusted least square means (LSM) ± standard errors (SE).

**Fig 6:**
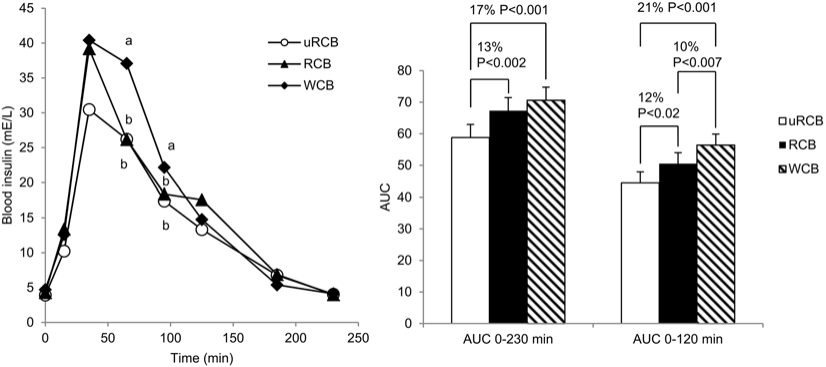
Plasma insulin profile and AUC. Left: Plasma insulin concentration- time profile in n = 23 participants. Values are means. A statistically significant interaction between treatment x time was detected (P<0.05). T-tests were performed for each time-point. Different letters indicate statistically significant differences between treatments at certain time points (P<0.05). Right: Differences in total AUC_0–230 min_ and AUC_0-120_ between breakfast treatments. Values are adjusted least square means (LSM) ± standard errors (SE). Differences between treatments are given as percentage difference between the LSM values for AUC of the treatments. P<0.05 was considered significant.

There was no significant difference in postprandial plasma glucose concentrations between any of the treatments (Fig 5). For insulin response, a significant treatment x time interaction was observed (P<0.05). Treatments differed significantly at certain time points and significant treatment differences were found when comparing AUC values (Fig 6). The response was significantly lower for uRCB and RCB compared with WCB at 65 and 95 minutes (P<0.05). Treatments differed significantly when comparing AUC_t = 0–230min_ (P<0.0001) and insulin secretion was 13% and 17% lower for uRCB compared with RCB (P<0.002) and WCB (P<0.001) respectively. There was no significant difference in AUC_t = 0–230min_ between RCB and WCB, but the insulin response for uRCB was 12% and 21% lower compared to RCB (P<0.02) and WCB (P<0.001), respectively. Treatments also differed significantly when comparing AUC_t = 0-120min_ (P<0.0001) and the insulin response was 10% lower for RCB compared with WCB (P<0.007).

### Microstructure

**Fig 7:**
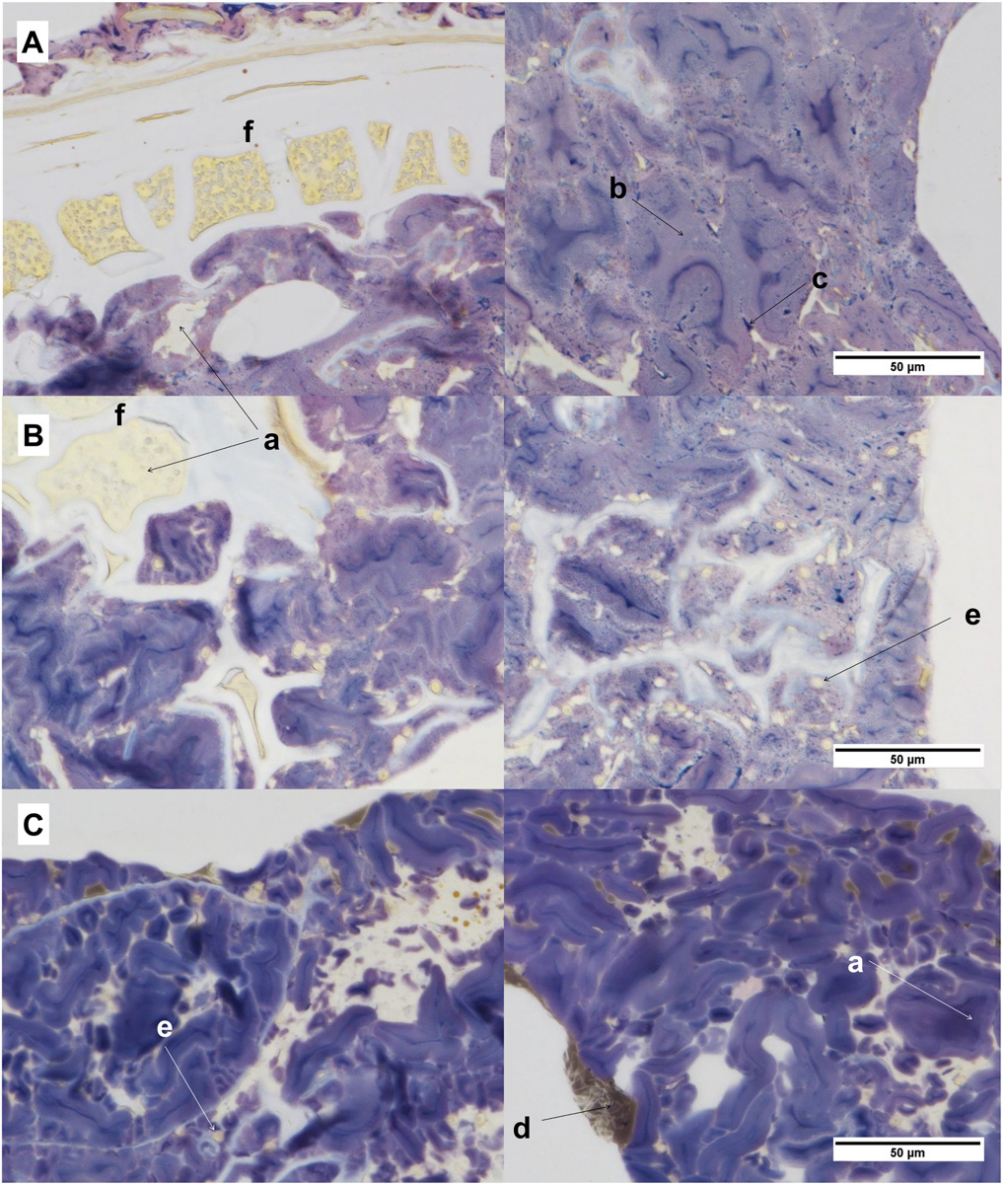
Micrographs showing the microstructure at the center of the crisp bread. (A) unfermented whole grain rye crisp bread (uRCB), (B) yeast-fermented whole grain rye crisp bread (RCB), and (C) yeast-fermented refined wheat crisp bread (WCB). Protein is colored yellow (a), amylopectin-rich areas purple (b), and amylose blue (c). Fat can be seen in WCB as brown aggregates (d) and yeast cells are present in WCB and RCB (e). Aleurone layers can be seen in uRCB and RCB (f).

The microstructure of the rye crisp breads differed from that of the wheat crisp bread in several respects. In uRCB and RCB (Fig 7B and 7C respectively), the continuous phase was composed of highly swollen and tightly packed starch granules with the protein packed into discrete aggregates. WCB (Fig 7A) was characterized by a continuous protein network and more intact and less swollen starch granules compared with uRCB and RCB. The difference in swelling of the starch granules was most clearly seen in samples from the center and top of the products. Starch granules closer to the surface facing down during baking exhibited less swelling in all products. Furthermore, in uRCB and RCB there was leakage of amylose, visualized as blue in the micrographs, which was concentrated in the center of swollen granules in all samples and between granules in the rye crisp breads. No difference could be seen between uRCB and RCB with regard to swelling of granules and leakage of amylose. Compared with WCB, uRCB and RCB contained more aleurone layers, encapsulating mainly protein, and some intact cells, encapsulating mainly starch. There also appeared to be a higher content of intact cells and aleurone layers in uRCB compared with RCB. Moreover, RCB and WCB contained yeast cells from the fermentation incorporated in the starch or protein matrix respectively. The white areas seen in the micrographs (Fig 7) are unstained material which represented pores in the crisp bread or fibers.

## Discussion

This study evaluated effects on appetite and postprandial glucose and insulin responses after consumption of unfermented whole grain rye crisp bread, fermented whole grain rye crisp bread and fermented refined wheat crisp bread as part of a complete breakfast. The rye crisp breads induced higher fullness and less hunger as well as lower insulin response postprandially than refined wheat crisp bread. Furthermore, the postprandial insulin response appeared to be different between fermented and unfermented crisp bread.

Intake of whole grain rye products in comparison with refined wheat bread has been shown to have an appetite-reducing effect in most studies where rye products such as porridge [6–8,11] and soft breads have been investigated [7,9,10,12,13]. A recent study by Forsberg et al [26] was the first to compare fermented whole grain rye crisp bread with refined wheat soft bread. They found, under similar conditions, that the rye crisp bread promoted 20–30% higher satiety and lower hunger and desire to eat compared with refined soft wheat bread. This is a larger effect than what we have found in the current study (11–16% with regard to fullness and hunger) where the control was refined wheat crisp bread. The energy density of products is known to influence postprandial responses such as satiety and glucose concentrations to some extent [24,25]. However, in this case the refined wheat crisp bread, which had a higher energy density than the refined wheat soft bread, resulted in a smaller difference in satiety compared with whole grain rye crisp breads. Thus other factors, such as composition and microstructural features of the products and differences between the study populations, are most likely of greater importance for the differences in postprandial responses between the studies. Further, we cannot exclude that the rather wide range in age could have diluted the differences between test meals with regard to hunger and insulin response.

Certain DF have been shown to have an appetite-reducing effect [37,38] and the content and composition of fiber may have contributed to the differences observed here in appetite response between the rye and wheat crisp breads. In particular viscous forming DF has been found to be efficient in reducing acute appetite responses [38]. The rye crisp breads tested here, uRCB and RCB, had a high content of DF (20.5% and 18.3% respectively) compared with the refined wheat crisp bread control (6% DF). The viscous forming arabinoxylan constituted 2.6%, 3.0% and 0.7% (extractable) and β-glucan 2.5%, 2.1% and 0.3% in uRCB, RCB and WCB respectively (Table 3). The mechanisms behind the high satiating capacity of DF are thought to be longer exposure time during mastication [39], increased gastric distension caused by water binding and decreased gastric emptying rate due to increased viscosity [40]. Moreover, the increased viscosity may reduce the diffusion rate of enzymes and nutrients in the digesta, thereby decreasing digestion and absorption rates [41,42].

The microstructural features of crisp bread products may also affect the absorption rate of nutrients. The rye crisp breads contained intact cells and fragments of aleurone layers, which were both absent in the refined wheat bread. These cells and fragments could lead to decreased digestion rate and lower digestibility of nutrients due to encapsulation [43,44], Lower digestibility of macronutrients in high-fiber rye products compared with refined wheat products with added cellulose fiber from grain straw (Vitacel) has recently been demonstrated in ileostomized human subjects [45]. As a consequence, a larger fraction of the nutrients in the rye products is likely to reach the distal ileum and the colon where they may contribute to the ileal break [46] and fermentation [47], respectively. uRCB appeared to contain more intact cells and larger fragments of aleurone layers than RCB, which may be due to the difference in particle size distribution of the flours used for baking. However, the difference between the flours was small in comparison with that used in studies reporting an effect of particle size on postprandial responses, and most likely had limited impact on the results [7,48].

The more swollen starch granules observed in the rye crisp breads would be expected to make the starch more susceptible to enzymatic degradation [49]. However, this may be counteracted by the presence of leaked amylose, as seen in both rye crisp breads, which has been suggested to form a protective layer around the starch granules, hindering enzymatic degradation [15]. Furthermore, in addition to the soluble fibers discussed above, a higher content of undigested material in the rye crisp breads, as a consequence of decreased digestion rate and digestibility, could, contribute to an increase in the viscosity of the digesta depending on size and amount of particles, influencing gastric emptying and intestinal transit rates [50].

When combined, the composition and content of DF and the microstructural properties of the rye crisp breads may also have contributed to a prolonged effect on gastric distension and release of satiety hormones [51], resulting in increased satiety, compared to refined wheat crisp bread.

Decreased rate of release and absorption of nutrients, e.g. glucose, would be expected to result in a corresponding decrease in concentration in the blood. However, all treatments in our study elicited similar glucose response but the rye crisp breads gave slightly lower insulin concentrations, which is in line with results from earlier studies [6–16,21]. The postprandial responses could have been influenced by the additional foods included in the breakfast, which may have obscured the actual effect on postprandial glucose and insulin concentrations of the rye and wheat crisp breads. However, the contribution with regard to nutrients was approximately the same, with a small difference in fat for WCB, for all treatments. Moreover, the results are in line with those of several studies in which no additional foods were included [10,12,13].

A recent study by Eelderink et al [52] showed that differences in the in vivo digestibility and absorption of starch are not necessarily reflected in blood glucose concentration. They suggested variations in the appearance of endogenous glucose or the glucose clearance rate as possible explanations. Higher postprandial insulin concentrations after intake of WCB could lead to a faster clearance rate of glucose from the blood circulation. Glucose could therefore have been absorbed faster from the small intestine after WCB compared with RCB and particularly uRCB, without being detected as a corresponding increase in blood concentration.

The higher content of viscous DF in the rye crisp breads could be expected to result in lower postprandial glucose responses compared with WCB. Decreased postprandial blood glucose concentrations have been shown for foods supplemented with different DF, and have been attributed to increased digesta viscosity [53–56]. In a recent study glucose concentration in the portal vein of pigs was measured after intake of refined wheat bread compared with rye bread and wheat bread supplemented with isolated arabinoxylan [57]. A trend for lower glucose absorption was seen at certain time points for both the rye bread and the arabinoxylan supplemented wheat bread. Both breads also elicited lower insulin responses than the refined wheat bread. However, another study on catheterized pigs only found an effect on insulin, and not on glucose absorption, when comparing whole grain wheat bread with bread with aleurone rye flour [58]. In that case the DF content was similar for both breads, which might have influence the results. The effect of breads supplemented with arabinoxylan has also been shown in humans, on glucose peak in one case [59] and on both glucose and insulin in another [60].

Fermentation of RCB most likely induced changes in the composition and content of DF compared with uRCB, which may also have influenced digesta viscosity and glucose absorption. Fermentation has been shown to reduce the content of fructans in RCB [61,62], and to cause degradation of the viscous arabinoxylan and β-glucan due to endogenous enzymes, mainly endoxylanases [63]. Rakha et al compared several fermented and unfermented whole grain rye crisp breads and found a shift towards lower molecular weight distributions in arabinoxylans and β-glucans after fermentation [22]. This may have an impact on the viscosity and consequently absorption rate of glucose and subsequent insulin response [64].

The differences in postprandial insulin response could also be due to different amounts of insulinogenic amino acids such as the BCAA valine, isoleucine and leucine as well as the amino acids lysine and threonine. These have been suggested to mediate increased secretion of insulin and to be correlated with higher concentrations of the incretins gastric inhibitory peptide (GIP) and glucagon-like peptide-1 (GLP-1) [19,65]. Higher concentrations of GIP and GLP-1 have also been seen postprandially, after ingestion of wheat bread in comparison with rye bread [15,16]. Furthermore, in studies comparing high fiber rye bread with refined wheat bread, lower plasma concentrations of isoleucine and leucine have been shown both in a single meal setting and after an eight-week intervention [20,21]. In the present study, the product content of isoleucine was 23% and 16% lower, and the content of leucine was 22% and 18% lower in uRCB and RCB, respectively, compared with WCB. The content of threonine and valine was approximately equal in all three crisp breads while lysine content was 34% and 16% higher in uRCB and RCB, respectively, compared to WCB. The difference in contents of BCAA in the crisp breads, may have contributed to the higher postprandial insulin response for WCB compared to uRCB and RCB. Moreover, the microstructural features discussed above are also likely to be important as the intact cell structures in the rye crisp breads would decrease the availability of BCAA in comparison with WCB. This may also differ slightly between the rye crisp breads due to the finer particle size of the flour in RCB, meaning more disrupted cells. Analysis of postprandial amino acid concentrations and incretine hormones may contribute to our understanding of the involvement of BCAA in regulation of the insulin response to wheat and rye products and is therefore warranted. Additionally, it may be of interest to investigate the role of bioactive compounds, such as benzoxazinoids and phenolic acids, in regulation of appetite and postprandial insulin and glucose responses [10,66,67]. The availability and absorption kinetics of these can be influenced by processing, e.g., yeast fermentation [68,69]. The extent to which bioactive compounds will contribute to the appetite regulation is still unknown.

## Conclusions

Unfermented (uRCB) and fermented (RCB) whole grain rye crisp bread as part of a complete breakfast induced higher satiety up to 4h after intake compared with refined wheat crisp bread (WCB). This difference was most likely due to higher content of dietary fiber, mainly viscous arabinoxylans and β-glucans, and microstructural features of the rye crisp breads.

Lower postprandial insulin concentrations, but no difference in glucose concentrations, were observed for both rye crisp breads compared with WCB and for uRCB compared with RCB. These differences in insulin responses may be due to altered glucose absorption kinetics caused by content- and process-induced changes in composition of dietary fiber in RCB and WCB, and higher content and availability of insulinogenic branched chain amino acids in WCB. Additionally, we cannot rule out effects of slight differences in particle size distribution.

## Supporting Information

S1 CONSORT Checklist Completed CONSORT checklist. (DOC)

S1 Dataset Raw data for glucose, insulin and subjective appetite ratings. (XLSX)

S1 Protocol Trial protocol. (DOCX)
