# Supplementary material for: Effects of Unfermented and Fermented Whole Grain Rye Crisp Breads Served as Part of a Standardized Breakfast, on Appetite and Postprandial Glucose and Insulin Responses: A Randomized Cross-over Trial
Source: PLoS One. 2015 Mar 31;10(3):e0122241. doi: 10.1371/journal.pone.0122241 (PMC4380355; doi:10.1371/journal.pone.0122241)
Supplement: S1 Protocol — (DOCX) [file pone.0122241.s003.docx]

**”Effekten av rågprodukter på aptit och metabolism”**

**”The effect of rye products on appetite and metabolism”**

***English translation (Google translation with minor necessary changes) of the Swedish trial protocol. Original Swedish protocol follows the English translation.***

**Research plan**

**Objective**
The overall objective of the study is to contribute to a better understanding of how dietary fiber in rye affects appetite and metabolic processes. We will evaluate how the intake of rye crisp bread affects self-rated appetite as well as insulin secretion and glucose levels in the blood of healthy adult humans in a meal study. We will also examine the microstructure of the products with microscopy, as well as and characterize the products with respect to dietary fiber and certain bioactive compounds (tocopherols and alkylresorcinols) to try to explain any differences in perceived appetite and insulin and glucose response. Satiety hormones will be analyzed (eg, GLP-1, GIP, PYY, CCK and ghrelin) and correlated with self-perceived satiety and microstructure to verify the findings from previous studies.

Specific primary goal of the study is:

- To conduct a short term intervention study in humans (n = 20) to evaluate the effect of high-fiber rye products on the self-perceived appetite by serving, as part of a breakfast, two kinds of fiber-rich rye bread (crisp breads) and compare against two white wheat flour bread (a soft and hard bread) as control.

Secondary objectives of the study:

- To evaluate the insulin and glucose response and investigate whether the plasma profiles of these are related to the perceived appetite. To help with this, bread samples will be characterized chemically and structurally.
- To evaluate the satiety hormones (GLP-1, GIP, PYY, CCK and grehlin) and related them to self-perceived saturation and microstructure.

**Background**
Lifestyle and diet are important factors in the prevention of chronic lifestyle diseases such as obesity, type 2 diabetes, cardiovascular disease and certain cancers (Smith & Tucker, 2011). A high intake of dietary fiber and fiber-rich foods such as whole grains, have in several studies been linked to a reduced risk of type 2 diabetes, cardiovascular disease and colon cancer, and in some studies even at reduced levels of biomarkers of inflammation in the blood (Landberg, 2012). Results from intervention studies in the short and medium term have shown effects on cholesterol levels, blood pressure, insulin secretion, glucose levels in the blood, satiety-egulating hormones and appetite (Bondia-Pons et al., 2011; Isaksson, 2011; Juntunen et al., 2003a, b; Leinonen et al., 1999; Rosen et al., 2009; 2011a, b, c; Wanders et al. 2011). However, the mechanisms in detail are largely unknown.

Glycemic index (GI) is a measure of the effect of food on the blood glucose level, where products with a low GI have been linked to health benefits by giving a slower and more balanced effect on glucose and insulin levels in the blood, allowing the oxidation of fat, reduce the formation of new fatty acids and lead to increased satiety (Kong et al., 2011). GI does not take into account fluctuations in blood glucose levels over time, but rather the total area under the glucose curve. This is unfavorable for foods, such as rye, which gives long and fairly low curves (Rosen et al., 2009). Rosen et al. (2009) introduced the glycemic profile (GP) as an alternative measure of how a food affects blood glucose profile following ingestion, which gives a better measure of glucose load after a meal, with for example rye, as the overall look of the curve is taken into account. Therefore, in this study GI* and GP will be studied for the products.

* GI is according to standard studied on foods with 50 g of available carbohydrate (Wolever et al., 2008). In this study, GI measurement is based on products with 25.5 g of available carbohydrates because the energy density of the included products is very low and that participants would otherwise have had to eat an unrealistically large amount of test products. GI measurements on crispbread with less than 50 g of available carbohydrate has previously made by Leinonen et al. (1999).

Several previous studies have observed lower insulin profile (IL, insulin index) in the blood after intake of rye compared with white wheat, but without the same effect on glucose profile (GI). The underlying reason for this phenomenon is not yet clear, but is believed to be linked to the specific microstructure of rye products in comparison to wheat products (Isaksson, 2011a). Additionally, soluble fiber, such as some arabinoxylans, is thought to, due to its water-binding capacity, result in higher viscosity and thus lower the speed of gastric emptying and absorption of carbohydrates from the small intestine (Rosen et al., 2011b). Products made ​​from rye have also been shown to result in a higher satiety both directly after meals and at subsequent goals. The microstructural characteristics and composition of foods, such as composition and properties of dietary fiber and starch content, fat and protein is critical for how the appetite, hormonal response and metabolism are affected by a meal. There is therefore great potential to optimize the properties of foods in order to get health benefits.

Rosen et al. (2011b) examined the effect of wholegrain rye bread on insulin and blood sugar levels as well as appetite, and found that all rye bread caused a higher perceived satiety than white bread and that the effect on insulin secretion and subsequent increased satiety and reduced desire to eat differed between loaves due to differences in content of fiber, starch and bioactive components, and hydrolysis of starch. Rosen et al. (2009) have also shown that the effect on insulin and glucose levels and perceived appetite also depends on what part of the rye used, where rye flour from whole grain and endosperm resulted in lower insulin levels and lower and slower glucose curves than bran. Also the contents of intact rye kernels gave increased satiety and reduced energy intake at subsequent lunch compared to bread consisting of the wheat kernels and rye bread without kernels (Rosen et al., 2011c). This has also been demonstrated by Isaksson (2011a, b). Rosen et al. (2011c) also confirmed that the bulking capacity of rye fiber, metabolites from fermentation in the colon, a high glycemic profile and a low insulin response likely contributes to the positive effects on appetite and glucose control in response to rye as seen in short- and medium-term time perspectives.

There are many different possible mechanisms behind the positive effects of dietary fiber on metabolic processes and saturation, and among these the microstructure and properties of the fiber have important roles (Lefranc-millot et al., 2009). Fibers provide an increased viscosity of the food during transport through the gastrointestinal tract resulting in a prolonged intestinal transit and a delayed absorption of nutrients such as glucose, which increases the feeling of satiety by enhancing satiety mediating signals to the central nervous system (Howarth et al., 2001). In addition, some dietary fiber is metabolized by intestinal bacteria to form metabolites which can affect glucose homeostasis and the feeling of satiety (Lefranc-millot et al., 2009). High-fiber foods generally have a low energy density, while taking longer to eat because they require more chewing, which increases the secretion of saliva and digestive juices. This contributes to increased satiety per calorie intake and thus perhaps also to better weight control (Howarth et al., 2010).

Short-term studies in healthy subjects have shown that rye products induce low insulin response and facilitate the regulation of glucose with lower peak levels of glucose in the blood and reduced risk of low blood sugar in the hours after a meal (Rosen et al., 2011a). Therefore, a diet rich in rye products is expected to contribute to a reduced risk of insulin resistance and oxidative stress, which are known risk factors for diet-related lifestyle diseases such as type 2 diabetes and cardiovascular disease (Rosen et al., 2011a). The glycemic pattern seen after a meal rich in rye is assumed to be the reason for the lower insulin secretion as rye products causes compared to wheat products and therefore it is of interest to study the glycemic profiles of rye products. The microstructure as well as the content, nature and composition of dietary fiber, starch and various organic acids have been shown to affect short- and long-term effects on glucose, insulin, and insulin secretion as well as the levels of inflammatory markers and perceived satiety (Holt & Miller, 1995; Juntunen et al., 2003a, b; Kristensen & Jensen, 2011; Pasman et al., 2003; Rosen et al. 2011a). For example, the fiber rich rye products have been shown to have interesting effects on glucose and insulin profiles after a meal, as well as on perceived satiety ( Isaksson, 2011a , b; Juntunen et al. 2003a , b; Rosen et al. 2011a , b, c ) .

**Importance for health**

It can be assumed that glycemic profile presented after a meal consisting of rye products such as rye bread is one reason for the reduced insulin demand, which have been linked to effects on appetite (Rosen et al., 2011b). Since increased satiety is a potential factor in preventing over-consumption of food, and thus a high energy intake with the risk of overweight and obesity, a diet containing fiber-rich rye products may work as a preventive factor for many lifestyle-related and metabolic diseases. Rye products with high fiber content have been associated with increased satiety and reduced hunger, and have been shown to affect blood profile of glucose and insulin after a meal by reducing the insulin response without a corresponding effect on glucose response. The microstructural characteristics and the composition of food products, such as dietary fiber, starch, fat and protein, are critical for how the appetite, hormonal response and metabolism are affected by a meal. There is therefore great potential to optimize the properties of foods in order to get health benefits. Thus, it is of interest to assess the effect on glycemic profile and perceived satiety of rye products, and try to link this to differences in chemical composition and structure of the products.

**Project hypothesis**

The project is based on the hypothesis that the whole grain rye crisp breads gives a greater perceived satiety than white crisp and soft white wheat bread. Two crisp breads with different fiber content will be tested. The primary hypothesis of the project is that a breakfast including rye crisp bread gives higher satiety than the wheat bread control (crisp bread and soft bread). The primary survey characteristic is self-assessed appetite registered using visual analogue rating scale, VAS. Rye has been previously shown to induce higher satiety compared to other cereals, and to influence the secretion of hormones and the flow of nutrients. Furthermore, we intend to investigate whether the effect on satiety is mediated by insulin, glucose and satiety hormones in the blood after ingestion of the test breakfasts. This will also be related to differences in structure and chemical nature between the rye crisp breads and the wheat breads.

**Project description**

**Subjects**

A total of 20 healthy men and women aged 18-70 years with a BMI of 18.5-30 (normal-obese) recruited through advertisements in local newspapers and on Ultuna Campus, Swedish University of Agricultural Sciences , Uppsala. Individuals interested in participating in the study will receive both verbal and written information about the study. A period of one week will be given for decision making after which the people who are still interested in participating will be recruited to the study. When recruiting, more detailed oral and written information about the study will be given, after which the research subjects give their consent to participate in the study. Thereafter a time is booked for screening of each participant.

A total of 30 people is to be screened at the clinic at the University Hospital in Uppsala. During the screening, research subjects’ height, weight and blood pressure will be measured and they will have to answer questions about their health, use of medications and herbal remedies, physical activity level and eating habits. The physical activity level (physical activity level, PAL) will be estimated by a short questionnaire and those with a very high physical activity level (PAL > 2.0) will be excluded. Cognitive and behavioral factors will be assessed by the revised Three- Factor Eating Questionnaire. Participants will be excluded if they have a value higher than 65% over the limit on one or more of the three categories: cognitive restraint, uncontrolled eating and emotional eating. Fasting blood samples will also be collected for analysis of glucose, insulin, thyroid stimulating hormone, hemoglobin, cholesterol and alaninaminostransferas (ALT). Only people whose values ​​are within the normal will be included in the study. If values ​​above or below normal are observed, the person concerned will be informed and urged to visit a doctor.

Only people who usually eat breakfast, lunch and dinner will be included in the study. Additional exclusion criteria are: diet, smoking and/or tobacco use, physiological and psychological problems related to eating, gastrointestinal problems or other medical conditions that are likely to affect appetite or food intake, pregnancy, lactation, or a desire to become pregnant during the study. Persons above 65 years will also be excluded as old age causes a decrease in food intake and faster perceived satiety, as well as children and young people as they may have difficulties to follow the study instructions.

**Test products**

Two commercial crisp breads baked with rye flour, a hard bread baked with white flour and a soft bread baked from white flour (the latter two products act as controls) will be included in the study.

Research subjects will after fasting be served the test breads (four test products, two rye breads and two white wheat bread as a control) as part of a standardized breakfast with margarine, ham and a small glass of juice and a choice of coffee and tea (the same drink and quantity shall be then ingested at all test occasions). Margarine will be used to make the test breakfasts isocaloric. The energy from the breakfasts will contribute with about 20 percent of the recommended daily energy intake for an average active person as recommended by the NFA. A total of four treatments will be carried out in a randomized, balanced cross-over design, with six days between so that any residual effects of a treatment will subside before the next treatment. Thus, each research subject will complete the study in just under five weeks' time, if no delays occur. With 20 participants, two research nurses, four treatments with a six-day normalization period, and 20 participants treated per week, the study will take approximately four to eight weeks to perform (not including any delays).

The test products will also be characterized chemically with respect to the content of dietary fiber (including fructans) and alkylresorsinoler, phenolic acids, cinnamic acids and tocopherols. While the products microstructure will be examined by light microscopy. This will be done at the Department of Food Science, SLU.

**Study design**

The intervention study will be performed in the Children's Hospital at Uppsala University Hospital (Clinical nutrition and metabolisms clinic). Research participants will be instructed to avoid food and drinks after 20:00 the day before participating in the study, and the last meal of the food should not contain fiber-rich foods such as whole grains, fruits and vegetables. Research subjects should not consume alcohol and refrain from strenuous physical activity for 24 hours preceding the test day.

On the morning of the test day participants should come to the clinic fasting before eating the test breakfast. Upon arrival, participants will be equipped with a hand held computer model Z22 (Palm Inc, Sunnyvale, CA, USA) for recording perceived appetite (satiety, hunger and desire to eat) on a visual analogue rating scale, VAS. This will be done every 30 minutes over a three hour period with the first registration just before the intake of breakfast. A trained research nurse will insert a peripheral venous catheter in research subjects left arm for analysis of insulin and glucose levels in the blood. Only trained nurses with sufficient experience will be used to minimize patient discomfort. Blood samples will be collected just before breakfast and at 15, 30, 45, 60, 90, 120, 150 and 180 minutes after breakfast (nine measurements), to analyze hormones, and glucose and insulin profiles and profiles of satiety hormones.

Immediately following the first appetite registration and the blood sample, breakfast will be served. Research subjects will eat separate from each other and eat within 15 minutes. They are not allowed to eat or drink anything out of the diet included in the study. This should be done in the same way during all study days. In total, the research subjects spend approximately three hours at the clinic on four different occasions. Research subjects are allowed to talk to each other, relax, read, etcetera with the exception that they should not discuss the study or compare their estimates.

**Analysis**
Insulin and glucose will be analyzed at the Department of Clinical Chemistry and Pharmacology, Uppsala University Hospital, Uppsala, Sweden. Satiety hormones will be analyzed by external expert laboratories. Chemical and structural characterization of the products will be performed at the Department of Food Science at the Swedish University of Agricultural Sciences, Sweden. Hormonal changes and metabolic changes will be related to the perceived appetite and differences in composition and structure of the test breads.

**Ethical considerations**

The food that is given in the study is considered to be a natural part of the Swedish normal diet. Because they contain a high percentage of natural fiber some may experience some mild discomfort with "noisy" stomach. This is usually temporary. Some discomfort/pain can be experienced when the venous cannula inserted into the arm. To minimize discomfort/pain experienced, only trained staff will put the cannula and take blood samples. The staff present at blood sampling is asked to report any unwished events to the principal investigators. The possible discomfort/pain participation may cause is judged to be very small in comparison to the value of the knowledge the results of the survey is expected to provide.

***Original Swedish trial protocol***

**Bilaga 2 Information om forskningsplan för fackmän**

**Vetenskaplig frågeställning**

Det övergripande målet med studien är att bidra till en ökad förståelse för hur kostfibrer i råg påverkar aptit och metabola processer. Vi kommer att utvärdera hur intag av rågknäckebröd påverkar självskattad aptit samt insulinutsöndring och glukosnivåer i blodet hos friska, vuxna människor i en måltidsstudie. Vi kommer även att undersöka mikrostrukturen hos produkterna med mikroskopi, liksom och karaktärisera produkterna med avseende på kostfibrer och vissa bioaktiva ämnen (tokoferoler och alkylresorcinoler) för att försöka förklara eventuella skillnader i upplevd aptit och insulin- och glukosrespons. Mättnadshormoner kommer att analyseras (t.ex. GLP-1, GIP, PYY, CCK och ghrelin) och korreleras till självupplevd mättnad och mikrostruktur för att verifiera fynd ifrån tidigare studier.

Specifika primära mål med studien är:

- Att genomföra en interventionsstudie på människa (n = 20) i ett korttidsperspektiv för att utvärdera effekten av fiberrika rågprodukter på den självupplevda aptiten genom att som en del av en frukost servera två sorters fiberrika rågbröd (knäckebröd) och jämföra mot två vita vetemjölsbröd (ett mjukt och ett knäckebröd) som kontroll.

Sekundära mål med studien:

- Att utvärdera insulin och glukossvaret och undersöka om plasmaprofilerna av dessa är relaterade till den upplevda aptiten. Som hjälp till detta kommer brödproverna att karaktäriseras kemiskt och strukturellt.
- Att utvärdera mättnadshormoner (GLP-1, GIP, PYY, CCK och grehlin) kommer att analyseras och relateras till självupplevd mättnad och mikrostruktur.

**Områdesöversikt**

Livsstil och kost är viktiga faktorer i förebyggandet av kroniska livsstilssjukdomar såsom fetma, diabetes typ 2 och hjärt- och kärlsjukdom och vissa cancerformer (Smith & Tucker, 2011). Ett högt intag av kostfibrer och fiberrika livsmedel såsom fullkornsprodukter, har i flera studier kopplats till minskad risk för diabetes typ 2, hjärt- och kärlsjukdom och tjocktarmscancer samt i vissa studier även till minskade halter av biomarkörer för inflammation i blodet (Landberg, 2012). Resultat från interventionsstudier på kort och medellång sikt har visat effekter på kolesterolnivåer, blodtryck, insulinutsöndring, glukosnivåer i blodet, mättnadsreglerande hormon och på aptit (Bondia-Pons *et* *al*., 2011; Isaksson, 2011; Juntunen *et al.*, 2003a,b; Leinonen *et al.,* 1999; Rosén *et al.*, 2009; 2011a,b,c; Wanders *et al.* 2011). Dock är mekanismerna i detalj till stor del okända.

Glykemiskt index (GI) är ett mått på livsmedels effekt på blodsockervärdet, där produkter med ett lågt GI har kopplats till positiva hälsoeffekter genom att ge en långsammare och mer balanserad effekt på glukos- och insulinnivåerna i blodet, tillåta oxidering av fett, minska nybildandet av fettsyror samt medföra ökad mättnad (Kong *et al.,* 2011). GI tar dock inte hänsyn till svängningar i blodsockernivåerna över tid utan enbart till den sammanlagda ytan under blodsockerkurvan vid ett intag, vilket missgynnar livsmedel med en lång och ganska låg kurva såsom råg (Rosén *et al.,* 2009). Rosén *et al.* (2009) introducerade glykemisk profil (GP) som ett alternativt mått på hur ett livsmedel påverkar blodsockerprofilen efter intag, vilket ger ett bättre mått på glukosbelastningen efter en måltid med exempelvis råg då kurvans totala utseende tas hänsyn till. I denna studie kommer därför GI* och GP att studeras för produkterna. *GI studeras enligt standard på livsmedel med 50 g tillgängliga kolhydrater (Wolever *et al*., 2008). I denna studie kommer GI-mätningen baseras på produkter med 25,5 g tillgängliga kolhydrater på grund av att energitätheten i de inkluderade produkterna är mycket låg och att deltagarna annars skulle behövt äta en orealistiskt stor mängd av testprodukterna. GI-mätningar på knäckebröd med mindre än 50 g tillgängliga kolhydrater har tidigare gjorts av Leinonen *et al.* (1999).

Flera tidigare studier har observerat lägre insulinprofil (IL, insulinindex) i blodet efter råg intag jämfört med vitt vete, dock utan samma påverkan på glukosprofilen (GI). Den bakomliggande orsaken till detta fenomen är ännu inte klarlagd, men tros vara kopplad till den den specifika mikrostruktur som rågprodukter har i jämförelse med veteproduckter (Isaksson, 2011a). Dessutom tros lösligafiber såsom vissa arabinoxylaner genom sin vattenbindande förmåga medföra högre viskositet och därmed sänka hastigheten på magtömningen och upptaget av kolhydrater från tunntarmen blir långsammare (Rosén *et al*., 2011b). Produkter gjorda av råg har även visats medföra en högre mättnad både direkt efter måltid och vid efterföljande mål. De mikrostrukturella egenskaperna och sammansättningen hos livsmedel, såsom sammansättning och egenskaper hos kostfiber och innehåll av stärkelse, fett och protein, är avgörande för hur aptit, hormonsvar och ämnesomsättning påverkas av en måltid. Det finns därför stor potential att optimera egenskaperna hos livsmedel i syfte att få positiva hälsoeffekter.

Rosén *et al.* (2011b) undersökte effekten av fullkornsrågbröd på insulinpåslag, blodsockernivåer och aptit och fann att alla rågbröd orsakade en högre upplevd mättnad hos deltagarna än vitt bröd och att effekten på insulinutsöndring och påföljande ökade mättnadskänslor och minskad önskan att äta skiljde mellan bröden beroende på skillnader i innehåll av fiber, stärkelse och bioaktiva komponenter samt hydrolys av stärkelse. Rosén *et al.* (2009) har även visat att effekten på insulinpåslag, glukosnivåer och upplevd aptit även beror på vilken del av rågen som används, där rågmjöl på fullkorn samt frövita ger lägre insulinpåslag och lägre och långsammare glukoskurvor än kli. Även innehållet av intakta? rågkärnor ger en ökad mättnad och ett lägre energiintag vid efterföljande lunch jämfört med bröd innehållandes vetekärnor och rågbröd utan kärnor (Rosén *et al.*, 2011c). Detta har även visats av Isaksson (2011a,b). Rosén *et al.* (2011c) bekräftade även att den bulkande kapaciteten hos rågfiber, metaboliter från fermentering i kolon, en hög glykemisk profil och ett lågt insulinsvar troligen bidrar till de positiva effekter på aptit- och glukosreglering som setts av råg i ett kort- och mellanlångt tidsperspektiv.

Det finns många olika möjliga mekanismer för hur kostfibrer kan ge positiva effekter på metaboliska processer och mättnad, och bland dessa spelar mikrostruktur och egenskaper hos fiber en viktig roll (Lefranc-millot *et al.*, 2009). Fibrer ger en ökad viskositet av födan vid transport genom mag- och tarmkanalen vilket medför en förlängd tarmpassage och en fördröjd absorption av näringsämnen såsom glukos, vilket ökar känslan av mättnad genom att förstärka mättnadsmedierande signaler till centrala nervsystemet (Howarth *et al.,* 2001). Dessutom kan vissa kostfibrer metaboliseras av tarmbakterier och bildar metaboliter vilka kan påverka glukoshomeostasen och känslan av mättnad (Lefranc-millot *et* *al*., 2009). Fiberrika livsmedel har generellt en låg energitäthet samtidigt som de tar längre tid att äta på grund av att de kräver mer tuggning vilket ökar utsöndring av saliv och magsafter. Detta bidrar till ökad mättnad per intagen kalori och därmed kanske även till bättre viktkontroll (Howarth *et al*., 2010).

Korttidsstudier på friska personer har visat att rågprodukter inducerar låga insulinpåslag och underlättar regleringen av glukos med lägre maximala nivåer av glukos i blodet och minskad risk för lågt blodsocker timmarna efter en måltid (Rosén *et al.*, 2011a). Därför kan en kost rik på rågprodukter antas bidra till en minskad risk för insulinresistens och oxidativ stress, vilket är kända riskfaktorer för kostrelaterade välfärdssjukdomar såsom diabetes typ 2 och hjärt- och kärlsjukdom (Rosén *et al.*, 2011a). Detta glykemiska mönster som ses efter en måltid rik på råg antas vara orsaken till den lägre insulinutsöndring som rågprodukter orsakar jämfört med veteprodukter och därför är det av intresse att studera rågprodukters glykemiska profil. Mikrostrukturen, innehållet, egenskaperna och sammansättningen av dietärt fiber, stärkelse och olika organiska syror har visats påverka kort- och långsiktiga effekter av glukos, insulin och insulinutsöndring såväl som nivåer av inflammatoriska markörer och upplevd mättnad (Holt & Miller,1995; Juntunen *et al.,* 2003a,b; Kristensen & Jensen, 2011; Pasman *et al.*, 2003; Rosén *et al.*, 2011a). Exempelvis så har fiberrika rågprodukter visats ha intressanta effekter på glukos- och insulinprofilerna efter en måltid, såväl som på upplevd mättnad (Isaksson, 2011a,b; Juntunen *et al.*, 2003a,b; Rosén *et al.*, 2011a,b,c).

**Betydelse för hälsa**

Det kan antas att den glykemiska profil som uppvisas efter en måltid bestående av rågprodukter såsom rågbröd är en orsak till det minskade insulinbehovet, vilket har kopplats till effekter på aptiten (Rosén *et al.*, 2011b). Eftersom ökad mättnad är en potentiell faktor för att förhindra överkonsumtion av mat och därmed ett högt energiintag med risk för övervikt och fetma, kan en kost innehållande fiberrika rågprodukter vara en förebyggande faktor för många livsstilsrelaterade och metabola sjukdomar. Rågprodukter med högt fiberinnehåll har kopplats till ökad mättnad och minskad hunger, samt har visats påverka blodprofilen av glukos och insulin efter måltid genom att minska insulinpåslaget utan motsvarande effekt på glukoskurvona. De mikrostrukturella egenskaperna och sammansättningen hos livsmedel, såsom kostfiber, stärkelse, fett och protein, är avgörande för hur aptit, hormonsvar och ämnesomsättning påverkas av en måltid. Det finns därför stor potential att optimera egenskaperna hos livsmedel i syfte att få positiva hälsoeffekter. Således är det av intresse att bedöma effekt på glykemisk profil och upplevd mättnad av rågprodukter, samt försöka koppla detta till skillnader i kemisk sammansättning och struktur hos produkterna.

**Projektets hypotes**

Projektet bygger på hypotesen att fiberrikt rågknäckebröd ger en större upplevd mättnad än vitt knäckebröd och mjukt vitt vetebröd. Två knäckebröd med olika fiberhalt testas. Den primära hypotesen i projektet är att en frukost inkluderande rågknäckebröd ger en högre mättnad än en vetebrödskontroll (knäcke- och mjukt bröd). Den primära undersökningsvariabeln är självskattad aptit registrerad med hjälp av visuell analog skattningsskala, VAS. Råg har i tidigare studier visat ge en högre mättnad jämfört med andra spannmål, samt att påverka insöndringen av hormoner och flödet av näringsämnen. Vidare avser vi att undersöka om den mättnadsgivande effekten medieras av insulin, glukos och mättnadshormoner i blodet efter intag av testfrukostarna samt. Detta kommer även att relateras till skillnader i struktur och kemisk karaktär mellan rågbröden och vetebrödet.

**Projektbeskrivning** *Forskningspersonerna*

Totalt kommer 20 friska män och kvinnor i åldern 18-70 år med ett BMI på 18,5 – 30 (normal- överviktiga) rekryteras genom annonsering i lokala nyhetstidningar och på Ultuna Campus, Sveriges lantbruksuniversitet, Uppsala. Personer som är intresserade av att delta i studien kommer att få både muntlig och skriftlig information om studien. En period på en vecka kommer att ges för beslutstagande efter vilken personer som fortfarande är intresserade av att delta kommer att rekryteras till studien. Vid rekrytering kommer mer ingående muntlig och skriftlig information om studien att ges, efter vilket forskningspersonerna ger sitt medgivande till att vara med i studien. Därefter bokas tid för screening för varje deltagare.

Totalt kommer 30 personer att screenas på kliniken vid Akademiska sjukhuset i Uppsala. Under screeningen kommer forskningspersonernas längd, vikt och blodtryck att mätas och de kommer att få svara på frågor angående sin hälsa, användning av läkemedel och naturläkemedel, fysisk aktivitetsnivå och matvanor. Den fysiska aktivitetsnivån (physical activity level, PAL) kommer att uppskattas genom ett kort frågeformulär och de med en mycket hög fysisk aktivitetsnivå (PAL>2.0) kommer att uteslutas. Kognitiva och beteenderelaterade faktorer kommer att bedömas genom reviderade Three-Factor Eating Questionnaire. Deltagare kommer att exkluderas om de får ett värde högre än 65 % över gränsvärdet på en eller fler av de tre kategorierna: kognitiv återhållsamhet, okontrollerat ätande eller känslomässigt ätande. Fasteblodprover kommer också att tas för analys av glukos, insulin, sköldkörtelstimulerande hormon, hemoglobin, kolesterol och alaninaminostransferas (ALAT). Endast personer vars värden ligger inom det normala kommer att inkluderas i studien. Om värden över eller under det normala observeras så kommer forskningspersonen i fråga att informeras och uppmanas att besöka läkare.

Endast personer vilka vanligtvis äter frukost, lunch och middag kommer inkluderas i studien. Ytterligare exklusionskriterier är: bantning, rökning och/eller snusning, fysiologiska och psykologiska problem att äta, mag- och tarmproblem eller andra medicinska tillstånd som sannorlikt riskerar att påverka aptit eller intag av mat, graviditet, amning eller en önskan att bli gravid under studiens gång. Personer över 65 år kommer också att exkluderas då hög ålder medför ett minskat matintag och snabbare upplevd mättnad, samt även barn och unga då de kan ha svårigheter att följa studiens instruktioner.

*Testprodukter*

Två kommersiella knäckebröd bakade på råg, ett knäckebröd bakat på vitt vetemjöl samt ett mjukt bröd bakad på vitt vetemjöl (varav de två senare produkterna fungerar som kontroller) kommer att ingå i studien.

Forskningspersonerna kommer efter fasta att serveras testbröd (fyra testprodukter; två rågbröd och två vita vetebröd som kontroll) som en del i en standardiserad frukost tillsammans med smörgåsmargarin och skinka samt ett litet glas juice och ett val mellan kaffe och te (samma dryck och mängd ska därefter intas vid alla testtillfällen). Smörgåsmargarin kommer att användas för att göra testfrukostarna isokaloriska. Frukostarnas energiintag kommer att bidra med ca 20 energiprocent för en medelaktiv person såsom rekommenderas av Livsmedelsverket. Totalt kommer fyra behandlingar att genomföras i en randomiserad och balanserad cross-over design, med sex dagar mellan så att eventuella kvarvarande effekter av en behandling hinner försvinna innan nästa behandling. Således deltar varje forskningsperson i studien i knappt fem veckors tid, om inga förseningar sker. Med 20 deltagare, två forskningssjuksköterskor, fyra behandlingar med en sex-dagars normaliseringsperiod, och 20 deltagare behandlade per vecka, kommer studien att ta uppskattningsvis fyra-åtta veckor att utföra (ej inkluderande eventuella förseningar).

Testprodukterna kommer också att karaktäriseras kemiskt med avseende på innehåll av kostfiber inklusive fruktaner samt alkylresorsinoler, fenolsyror, kanelsyror och tokoferoler. Även testprodukternas mikrostruktur kommer att undersökas med mikroskopi. Detta kommer att ske på Institutionen för livsmedelsvetenskap, SLU.

*Studiedesign*

Interventionsstudien kommer att utföras i vid Barnsjukhuset vid Akademiska Sjukhuset i Uppsala (Klinisk nutritions och metabolisms klinik). Forskningsdeltagarna kommer att instrueras att dagen innan deltagande i studien undvika mat och dryck efter klockan 20.00, och att det sista målet mat inte bör innehålla fiberrika livsmedelsprodukter såsom fullkornsprodukter och frukt och grönsaker. Forsknings-personerna bör inte heller konsumera alkohol och bör avstå från ansträngande fysisk aktivitet under 24 timmar föregående testdagarna.

På morgonen till testdagarna ska forskningsdeltagarna komma fastande till kliniken för att äta testfrukosten. Vid ankomst kommer deltagarna att utrustas med en handburen dator model z22 (Palm Inc, Sunnyvale, USA), för att elektroniskt registrera upplevd aptit (mättnad, hunger och önskan at äta) på en visuell analog skattningsskala, VAS. Detta kommer göras var 30e minut under en tretimmarsperiod med första registreringen precis innan intag av frukost. En tränad forskningssjuksköterska kommer att sätta en perifer venkateter i forskningspersonernas armveck för analys av insulin- och glukosnivåer i blodet. Endast utbildade sjuksköterskor med tillräcklig erfarenhet kommer att användas för att minimera patientens obehag. Blodprover kommer att insamlas precis innan frukost samt vid 15, 30, 45, 60, 90, 120, 150 och 180 minuter efter frukost (totalt nio mätningar), för att kunna analysera hormoner och glukos- och insulinprofiler samt profiler av mättnadshormoner.

Direkt efter den förta aptitregistreringen och blodprovet kommer frukosten att serveras. Forskningspersonerna kommer att äta skilda från varandra och äta upp inom 15 minuter. Det är inte tillåtet att äta eller dricka något utöver den kost som ingår i studien. Detta ska göras på samma sätt under alla studiedagar. Totalt kommer forskningspersonerna spendera uppskattningsvis tre timmar på kliniken vid fyra olika tillfällen. Forskningspersonerna är tillåtna att prata med varandra, koppla av, läsa et cetera med undantaget att det inte bör diskutera studien eller jämföra sina skattningar.

*Analys*

Insulin och glukos kommer att analyseras på Avdelningen för Klinisk Kemi och Farmakologi på Uppsala Universitetssjukhus, Uppsala, Sverige. Mättnadshormoner kommer att analyseras av externa expertlaboratorier. Kemisk och strukturell karaktärisering av produkterna kommer att utföras på institutionen för livsmedelsvetenskap på Sveriges lantbruksuniversitet, Ultuna, Sverige. Hormonella förändringar och metaboliska förändringar kommer att relateras till upplevd aptit och till skillnader i sammansättning och struktur hos testbröden.

**Etiska överväganden**

De livsmedel som ges i studien är att betrakta som en naturlig del av den svenska normalkosten. Eftersom de innehåller en hög andel naturliga kostfibrer så kan vissa uppleva milda besvär med "bullrig" mage. Detta är oftast övergående. Ett visst obehag/smärta kan upplevas då venkanylen sätts i armvecket. För att minimera obehag/smärta är det erfaren och utbildad personal som sätter kanylen och tar blodproven. Personalen vid blodprovstagningen ombeds att rapportera eventuella oönskade händelser till ansvariga forskare. Det eventuella obehag/smärta ett deltagande kan medföra bedöms som mycket ringa i förhållande till kunskapsvärdet av de resultat som undersökningen förväntas ge.

**Nyckelreferenser**

Bondia-Pons, I., Nordlund, E., Mattila, I., Katina, K., Aura, A.-M., Kolehmainen, M., Orešič, M., et al. (2011). Postprandial differences in the plasma metabolome of healthy Finnish subjects after intake of a sourdough fermented endosperm rye bread versus white wheat bread. *Nutrition journal*, *10*(1), 116. doi:10.1186/1475-2891-10-116

Butera, P. C. (2010). Estradiol and the control of food intake. *Physiology & behavior*, *99*(2), 175–80. doi:10.1016/j.physbeh.2009.06.010

Holt, S. H., & Miller, J. B. (1995). Increased insulin responses to ingested foods are associated with lessened satiety. *Appetite*, *24*(1), 43–54. Retrieved from http://www.ncbi.nlm.nih.gov/pubmed/7741535

Howarth, N. C., Sc, M., Saltzman, E., Roberts, S. B., & Ph, D. (n.d.). Lead Review, *59*(5).

Isaksson, H. (2011). *Satiating Effects of Rye Foods*.

Isaksson, H., Rakha, A., Andersson, R., Fredriksson, H., Olsson, J., & Aman, P. (2011). Rye kernel breakfast increases satiety in the afternoon - an effect of food structure. *Nutrition journal*, *10*(1), 31. doi:10.1186/1475-2891-10-31

Juntunen, K. S., Laaksonen, D. E., Autio, K., Niskanen, L. K., Holst, J. J., Savolainen, K. E., Liukkonen, K.-H., et al. (2003). Structural differences between rye and wheat breads but not total fiber content may explain the lower postprandial insulin response to rye bread. *The American journal of clinical nutrition*, *78*(5), 957–64. Retrieved from http://www.ncbi.nlm.nih.gov/pubmed/14594782

Juntunen, K. S., Laaksonen, D. E., Poutanen, K. S., & Niskanen, L. K. (2003). High-fiber rye bread and insulin secretion and sensitivity in healthy.

Kristensen, M., & Jensen, M. G. (2011). Dietary fibres in the regulation of appetite and food intake. Importance of viscosity. *Appetite*, *56*(1), 65–70. doi:10.1016/j.appet.2010.11.147

Landberg, R. (2012). Dietary fiber and mortality : convincing observations that call for, 3–4. doi:10.3945/ajcn.112.040808.Am

Landberg, R., Andersson, S., Zhang, J., Johansson, J., Adlercreutz, H., & Kamal-eldin, A. (2010). Rye Whole Grain and Bran Intake Compared with Refined Wheat Decreases Urinary C-Peptide , Plasma Insulin , and Prostate Specific Antigen in Men with Prostate Cancer 1 – 3. doi:10.3945/jn.110.127688.radical

Lefranc-millot, C., Macioce, V., Guérin-deremaux, L., Lee, A. W., & Cho, S. S. (2009). Fiber and Satiety 8.

Leinonen, K., Liukkonen, K., Poutanen, K., Uusitupa, M., & Mykkänen, H. (1999). Rye bread decreases postprandial insulin response but does not alter glucose response in healthy Finnish subjects. *European journal of clinical nutrition*, *53*(4), 262–7. Retrieved from http://www.ncbi.nlm.nih.gov/pubmed/10334650

Pasman, W. J., Blokdijk, V. M., Bertina, F. M., Hopman, W. P. M., & Hendriks, H. F. J. (2003). Effect of two breakfasts, different in carbohydrate composition, on hunger and satiety and mood in healthy men. *International journal of obesity and related metabolic disorders journal of the International Association for the Study of Obesity*, *27*(6), 663–668. doi:10.1038/sj.ijo.0802284

Rosén, L. (2011). *METABOLIC PROPERTIES OF RYE regulation in healthy subjects*.

Rosén, L. a H., Silva, L. O. B., Andersson, U. K., Holm, C., Ostman, E. M., & Björck, I. M. E. (2009). Endosperm and whole grain rye breads are characterized by low post-prandial insulin response and a beneficial blood glucose profile. *Nutrition journal*, *8*, 42. doi:10.1186/1475-2891-8-42

Rosén, L. a H., Östman, E. M., & Björck, I. M. E. (2011a). Postprandial glycemia, insulinemia, and satiety responses in healthy subjects after whole grain rye bread made from different rye varieties. 2. *Journal of Agricultural and Food Chemistry*, *59*(22), 12149–54. Retrieved from http://www.ncbi.nlm.nih.gov/pubmed/21981244

Rosén, L. a H., Östman, E. M., & Björck, I. M. E. (2011b). Postprandial glycemia, insulinemia, and satiety responses in healthy subjects after whole grain rye bread made from different rye varieties. 1. *Journal of Agricultural and Food Chemistry*, *59*(22), 12149–54. Retrieved from http://www.ncbi.nlm.nih.gov/pubmed/21981244

Rosén, L. A., Östman, E. M., & Björck, I. M. (2011). Effects of cereal breakfasts on postprandial glucose, appetite regulation and voluntary energy intake at a subsequent standardized lunch; focusing on rye products. *Nutrition Journal*, *10*(1), 7. doi:10.1186/1475-2891-10-7

Smith, C. E., & Tucker, K. L. (2011). Health benefits of cereal fibre: a review of clinical trials. *Nutrition Research Reviews*, *24*(1), 118–131. doi:10.1017/S0954422411000023

Wolever, T. M. S., Brand-miller, J. C., Abernethy, J., Astrup, A., Atkinson, F., Axelsen, M., Björck, I., et al. (2008). Measuring the glycemic index of foods : interlaboratory study 1 – 5, *87*.
